# A first glimpse into circulating ghrelin patterns of thin-billed prion chicks (*Pachyptila belcheri*)

**DOI:** 10.1007/s00360-025-01602-7

**Published:** 2025-02-14

**Authors:** Julia Slezacek, Leonida Fusani, Hiroyuki Kaiya, Petra Quillfeldt

**Affiliations:** 1 Department of Interdisciplinary Life Sciences, Konrad Lorenz Institute of Ethology, University of Veterinary Medicine Vienna, Savoyenstraße 1A, 1160 Vienna, Austria; 2 Department of Cognitive Biology, University of Vienna, Djerassiplatz 1, 1030 Vienna, Austria; 3 Department of Biochemistry, National Cerebral and Cardiovascular Center Research Institute, 6-1 Kishibe-Shinmachi, Suita, 564-8565 Japan; 4 Research Division of Drug Discovery, Grandsoul Research Institute for Immunology, Inc., 8-1 Utano-Matsui, Uda, Nara 633-2221 Japan; 5 Faculty of Science, University of Toyama, 3190 Gofuku, Toyama-City, Toyama 930-8555 Japan; 6 Department of Animal Ecology & Systematics, Justus Liebig University Giessen, Heinrich-Buff-Ring 26, 35392 Giessen, Germany

**Keywords:** Ghrelin, Chick development, Fasting, Food intake, Thin-billed prion, *Pachyptila belcheri*

## Abstract

The peptide hormone ghrelin, also known as “hunger hormone”, is primarily secreted by the stomach and plays a key role in the regulation of vertebrate appetite and energy balance. While the hunger hormone and its functions have been extensively researched in mammalian species, its physiological roles have received less attention in birds and knowledge on the ghrelin system is especially poor in wild avian species. In contrast to mammals, ghrelin acts as an anorexigenic signal in birds and suppresses food intake. In this study, we focussed on the altricial chicks of thin-billed prions (*Pachyptila belcheri*) which are subjected to irregular, up to 8 day-long fasts, while waiting for their parents to return from feeding trips. We show that thin-billed prion chicks, which received a meal in the night prior to sampling, had higher circulating ghrelin levels than fasting conspecifics. Ghrelin levels did not correlate with chick body condition, meal size, or the length of a fast. Our study adds to past literature supporting an anorexigenic effect of avian ghrelin and is among the first to describe ghrelin profiles in seabirds, thereby significantly contributing to the scarce literature on ghrelin in wild avian species.

## Introduction

Ghrelin is a peptide hormone mainly produced in the stomach, signalling hunger via the ghrelin receptor GHSR-1a (reviewed in Müller et al. 2015). In mammals, circulating ghrelin concentrations rise during fasting and peak in anticipation of a meal, and the hormone reflects lipid stores and promotes weight gain (reviewed in e.g. Cummings 2006; Álvarez-Castro et al. 2013). Following the ingestion of a meal, ghrelin levels typically decrease again and circulating ghrelin concentrations were found to be linked to metabolic diseases such as obesity, where obese individuals were found to have lower levels of the hormone (reviewed in Álvarez-Castro et al. 2013). Due to these characteristics, the “hunger hormone” has rapidly moved into the spotlight of numerous medical studies. As much as the role of ghrelin in mammalian feeding regulation is undebatable, its function and role in appetite regulation remain largely understudied in other vertebrate classes, such as birds. In birds, ghrelin was shown to inhibit food intake thus working as an anorexigenic signal opposed to the orexigenic function in mammals (reviewed in Kaiya et al. 2007a). Literature on ghrelin in wild birds remains characterised by a multitude of open questions as the hormone has only recently received attention outside the poultry industry. Studies on different passerine species found positive relationships between ghrelin and internal energy stores during migration but a negative effect of ghrelin on food hoarding and body mass gain in a wintering songbird (Goymann et al. 2017; Lupi et al. 2022; Henderson et al. 2018). Yet, these results could not always be confirmed (Eikenaar et al. 2018; Williamson et al. 2024). Importantly, a new study investigating the genetic background of avian ghrelin highlights a loss of the ghrelin gene in most passerine species and the authors postulate that the intact ghrelin receptor may be activated by another still unknown hormone (Prost et al. 2023). Seim and colleagues (2015) further show a loss of ghrelin in the saker (*Falco cherrug*) and peregrine falcon (*Falco peregrinus*), however, they highlight ghrelin to be intact in a range of other non-passerine species (Seim et al. 2015). A recent study of our team on southern rockhopper penguins (*Eudyptes chrysocome chrysocome*) found circulating ghrelin profiles to be differently regulated between moulting (fasting) and non-moulting (feeding) adults. Furthermore, we found ghrelin levels to be significantly lower in chicks compared to adults (Slezacek et al. 2024). Undeniably, recent years have witnessed a rise in studies aiming at uncovering the regulation of feeding in wild birds in different stages of the life cycle and resulting varying energetical needs, however, the above-mentioned studies underline the remaining vast knowledge-gap on a hormone known to play a major role in feeding regulation in vertebrates.

In this short communication we aim at widening the limited knowledge on ghrelin in wild, non-passerine avian species and report results of work investigating circulating ghrelin of chicks of the wild thin-billed prion (*Pachyptila belcheri*), a pelagic *Porcellariiform* bird species which breeds on islands of the Southern Ocean, such as Kerguelen, the Falklands, Isla Noir (Chile), and Crozet (Marchant and Higgins 1990). Chicks of thin-billed prions are altricial and fully dependent on food provisions by their parents wherefore adults embark on long feeding trips, often leaving their chicks unattended and unfed for several days. This means that chicks rarely receive food in regular intervals and need to cope with periods of complete fasting until the next feeding bout (Quillfeldt et al. 2007a). The chicks remain in the same nesting burrow until fledging, facilitating the monitoring of feeding intervals and body condition of individual animals. Taken together, these characteristics make thin-billed prion chicks a suitable wild study system in the research of interactions between hormones and feeding/ fasting states (e.g. Quillfeldt et al.^2006^, 2007b, 2009). The objective of this study was describing ghrelin patterns of thin-billed prion chicks and testing whether feeding events or body condition influence the circulating concentrations of the hormone.

## Material and methods

We conducted sample collection in February 2019 on New Island (51°43´ S, 61°17´ W), the most important breeding site for thin-billed prions (*Pachyptila belcheri*) in the Falkland Islands (Catry et al. 2003). Thin-billed prions are medium-sized prions with a mean adult body mass of 130 g. They are nocturnal, burrow-nesting birds and typically lay one egg into a shallow nesting chamber. The average fledging time of the fully altricial chicks is ca. 50 days and both parents provide their offspring with food during chick-development (Quillfeldt et al. 2010). For that, breeding adults embark on feeding trips, often flying long distances to find suitable marine resources (Quillfeldt et al. 2022). This means that chicks sometimes remain several days without feeding until parents return with food.

At our study site, nesting chambers are reachable through artificial tunnels (perpendicular to the natural entrance tunnel) to allow a fast access to chicks, resulting in short handling times, and causing minimal disturbance (Quillfeldt et al. 2007a). We collected samples from 38 – 40-day old chicks, which either had been recently provided with a meal or had been fasting for 1 – 8 days. Sampling was conducted between 10:00 and 22:00 when parents were not present at burrows. For blood sampling we punctured the brachial vein with a sterile needle and used heparinized microcapillary tubes to draw a maximum of 200 µl of whole blood, which we collected within 3 min to minimize handling stress. We stored samples in insulated cooled bags until centrifuging them for 5 min (2700 rcf) to extract plasma. We added 1 M HCl (1:10 HCl:plasma), and stored samples in liquid nitrogen until arrival to our laboratory in Vienna where they were transferred to a -80 °C freezer. After blood sampling we weighed chicks (to the nearest g) using a digital balance and measured wing length (to the nearest mm) using a wing ruler. If chicks were already present on our first visit, we determined their hatching date (to the nearest day) by calibrating wing length against wing growth in chicks of known age. We weighed and measured chicks daily and estimated meal sizes and feeding rates after Quillfeldt et al. (2003), by correcting mass differences for metabolic mass loss, adapted for one daily measurement. We estimated chick body condition from daily body mass, after Quillfeldt et al. (2007c): We calculated an index of chick body condition relative to the mean mass for study chicks of each age.

Ghrelin concentration in plasma was measured at the National Cerebral and Cardiovascular Center Research Institute (Osaka, Japan) via radioimmunoassay (RIA). We have successfully used this method previously for wild non-galliform species (e.g. Slezacek et al. 2024) and a detailed RIA description can be found in Kaiya et al. (2007b). Samples were measured in one assay and intra-assay variance amounted to less than 5%.

We performed statistical analyses in R Studio (2024.4.1.748; Posit team 2024). We used separate ANOVAs for testing possible correlations between ghrelin levels and feeding status, meal size, length of fasting (unfed chicks only), body condition, and daily body mass change. We checked each ANOVA fit using diagnostic plots in base R.

## Results

**Fig. 1:**
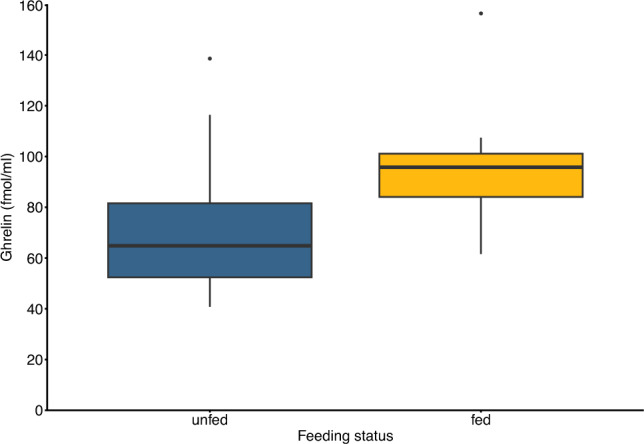
Circulating ghrelin concentrations between thin-billed prion chicks that had been fed in the night preceding sampling (fed; yellow box; n = 9) compared to chicks that had not received a meal (unfed; blue box; n = 13). Fed chicks had significantly higher ghrelin levels (p = 0.04). Lines within boxes represent median ghrelin concentrations and ends of whiskers represent highest and lowest values within 1.5 * interquartile ranges (IQR). Points above boxes represent two statistical “outliers” that did not fall into 1.5 * IQR. The points were not excluded from statistical analyses due to there being neither biological nor methodological reason for an exclusion

We found significantly higher plasma ghrelin concentrations in chicks that had been fed by their parents in the night before sampling compared to chicks that had not received a recent meal (*F*_*(1,20)*_ = 4.82, *p* = 0.04, η2 = 0.19; Fig. 1). The mean ghrelin concentration measured in chicks which had received a meal was 104.82 fmol/ml and in birds that had not received a meal 74.18 fmol/ml. The estimated size of the meal did not influence measured ghrelin concentrations (*F*_*(1,7)*_ = 0.02, *p* = 0.89, η2 < 0.01). Ghrelin levels did not differ between chicks that were fasted for one night and chicks that were fasted two or more nights (*F*_*(1,11)*_ = 0.29, *p* = 0.60, η2 = 0.03). Plasma ghrelin concentrations were further not correlated with individual body condition (*F*_*(1,20)*_ = 0.62, *p* = 0.44, η2 = 0.03) or daily body mass change (*F*_*(1,20)*_ = 1.77, *p* = 0.20, η2 = 0.08). We measured a mean body mass of 179.67 g (range: 140–208 g) in chicks that had received a meal and a mean body mass of 158.15 g (range: 93–238 g) in chicks that were not fed.

## Discussion

We here present a first report of circulating ghrelin in wild thin-billed prion chicks. We show that chicks fed prior to sampling had higher plasma ghrelin concentrations compared to chicks that had not been fed (Fig. 1). Ghrelin levels were not associated with body condition or the length of fasting. We further could not detect a relationship between meal size and ghrelin in recently fed individuals.

Research on poultry shows that ghrelin acts as a feeding-restricting hormone in birds (reviewed in Kaiya et al. 2013a, b). Our result of thin-billed prion chicks having higher circulating ghrelin concentrations after a recent meal, compared to lower concentrations when fasted, add to literature confirming that ghrelin levels increase after ingestion of food in birds. Our results are however not in line with our previous study in a wild, non-galliform species, where we did not detect any difference in ghrelin levels between fed and unfed chicks of southern rockhopper penguins (Slezacek et al. 2024). A contrasting pattern of ghrelin dynamics was found in fasted and fed chicks of layer chicken (*Gallus gallus domesticus*), with chicks having increased plasma ghrelin concentrations after a 12 h fast (Kaiya et al. 2007b). Yet, another study on fed and fasted chicks of broad-breasted white turkeys (*Meleagris gallopavo*) found the same pattern as we report in thin-billed prions (Vizcarra et al. 2018). The differing results between chicken and turkey could be caused by species-specificity of ghrelin dynamics and additionally, by a difference in ghrelin amino acid sequences at the carboxyl-terminal of the hormone precursor (Vizcarra et al. 2018). Yet, the sharp contrasts between chick development and the physiological changes caused by domestication and the food industry, complicate the comparison of data stemming from experiments on domesticated poultry vs. wild species. One main difference between the study on penguins and the present one is the elapsed time between feeding and sampling events, with blood sampling in the penguin study having occurred close to chick feeding (15 min post feeding), whereas in the present study, sampling occurred between midday and evening after chicks were fed. We think it likely that the time between feeding and sampling contributes to the differing results between penguins and prions as research in mammals has shown that although ghrelin changes are initiated by food intake, peak changes occur one to two hours after feeding (Cummings et al. 2001; Takahashi et al. 2012). Furthermore, a larger scale comparison of the genetic background of avian ghrelin protein sequences is required to understand whether possible differences in genetics influence ghrelin dynamics. We hope future studies on avian ghrelin will focus on a wider range of wild altricial and precocial species to establish if and how developmental strategies or phylogeny influence ghrelin patterns. Additionally, the possible effect of elapsed time between food intake and sampling should be considered and it is important to establish time curves for ghrelin dynamics in a variety of species pre- and post-feeding (*i.e.* when do ghrelin levels peak/ bottom, how long do levels rise/ decline in response to feeding/ fasting).

Recently, Marasco and colleagues (2023) found ghrelin to reflect body condition in migratory common quails (*Coturnix coturnix*) and Shousha et al. (2015) showed that ghrelin administration decreased body mass gain in Japanese quails (*Coturnix japonica*). We did not find a relationship between plasma ghrelin and body condition in thin-billed prions. The studies on quails were both performed in adult individuals and past research on avian ghrelin has found ghrelin patterns to differ according to developmental stage (Yamato et al. 2005; Yu et al. 2016; Wang et al. 2009; Slezacek et al. 2024). In future research it will be interesting to elucidate whether a ghrelin and body condition link might be influenced by developmental stages.

We did not detect relationships between ghrelin and the length of fasting or between ghrelin and meal size. The sample size in our study was limited because of the nature of the study population and we had therefore few datapoints available for different meal sizes or duration of fasting. To make accurate comparisons, it will be crucial to collect more data from chicks having received large vs. small meals and individuals with shorter vs. longer fasts as this will increase the power of statistical tests.

In conclusion, our study on thin-billed prion chicks reports higher circulating ghrelin levels after a feeding event compared to conspecifics which did not receive food, confirming past literature showing avian ghrelin to increase after meals, which suggests an anorexigenic action of this hormone. Yet, the data presented here answer only a fraction of the open questions on the avian ghrelin system, and future studies should focus on ghrelin dynamics in different developmental and life cycle stages, the hormone’s interplay with other endocrine and physiological factors, and its genetic background in a larger array of species.

## Data Availability

Raw data is available in the public online repository Zenodo: 10.5281/zenodo.14609428.
